# Psychometric properties of the Chinese version of the Obsessive Beliefs Questionnaire-44 (OBQ-44)

**DOI:** 10.1186/s12888-015-0579-6

**Published:** 2015-08-04

**Authors:** Jing Wang, Zhen Wei, He Wang, Zeyu Jiang, Ziwen Peng

**Affiliations:** Centre for Studies of Psychological Application, School of Psychology, South China Normal University, 55 Zhongshang Road, Guangzhou, 510630 China; Shenzhen Maternal and Child Healthcare Hospital, Shenzhen, 518028 China; Guangzhou Psychiatry Hospital, Guangzhou, 510370 China

## Abstract

**Background:**

The Obsessive Beliefs Questionnaire-44 (OBQ-44) is originally developed by the Obsessive Compulsive Cognitions Working Group and has been translated into several languages. This paper is aimed to investigate the psychometric properties of the Chinese version of the Obsessive Beliefs Questionnaire-44 (OBQ-44) in both clinical and non-clinical samples.

**Methods:**

Five hundred and sixty-nine undergraduate volunteers and sixty-six OCD patients were included in the study. All participants have completed Chinese version of OBQ-44, Yale-Brown Obsessive-Compulsive Scale (Y-BOCS), and Beck Depression Inventory (BDI). Confirmatory factor analysis was conducted to examine the construct validity of Chinese version of OBQ-44. The internal consistency and test-retest reliabilities at 4-week interval were examined in both non-clinical and clinical groups.

**Results:**

The confirmatory factor analysis of the non-clinical sample confirmed a 3-factor model which was suggested by the original authors of the instrument (*χ*^2^/d.f = 2.96, GFI = 0.83, NFI = 0.82, CFI = 0.88 and RMSEA = 0.06). The internal consistency and test-retest reliability were at an acceptable range for the two samples.

**Conclusions:**

The Chinese version of OBQ-44 is a valid and reliable instrument for assessing dysfunctional beliefs related to the etiology and maintenance of obsessions and compulsions.

## Background

Obsessive-compulsive disorder (OCD) is characterized by the presence of obsessions and/or compulsions that are time-consuming and usually provoke distress [1]. A considerable body of evidence from both cross-sectional [2, 3] and interventional research [4] has emerged to support the proposed theory that dysfunctional beliefs play a core role in the etiology and maintenance of obsessions and compulsions. Adequate recognition of specific dysfunctional belief domain associated with OCD and a regular monitoring of changes in dysfunctional beliefs is essential to guide appropriate treatments of OCD patients. Therefore, it is important to develop reliable instruments with tested validity in different populations from different countries and cultures for recognizing specific dysfunctional belief domain associated with OCD. The forty-four items version of Obsessive Beliefs Questionnaire (OBQ-44) was originally developed by Obsessive Compulsive Cognitions Working Group (OCCWG, 2005) to assess dysfunctional beliefs related to the etiology and maintenance of obsessions and compulsions [5]. The OBQ-44 is a self-report based instrument that contains three sub-scales: responsibility/threat estimation (RT), perfectionism/certainty (PC), and importance/control of thoughts (ICT). Sub-scales scores can provide more detailed information about dysfunctional belief domains associated with OCD, which is important to study the pathophysiology of dysfunctional beliefs in OCD. For example, Nakamae et al. pointed there was a significant negative correlation between gray matter volume and OBQ-ICT scores in the left amygdale which may play a role in the presence of certain dysfunctional beliefs in OCD patient [6]. The OBQ-44 was effective to conduct a comprehensive evaluation of dysfunctional beliefs in OCD patients because it is scored on a 7-point Likert scale (1 to 7) which represents different levels of severity. The OBQ-44 has been translated into several languages and has validated clinical and non-clinical samples [7–11]. Furthermore, OBQ-44 was shown to have good psychometric properties in both clinical and non-clinical samples in different language versions. However, most of these validations were done in western samples. Its reliability and validity in non-western population were still limited, and its psychometric properties need further research. Moreover, to our knowledge, there is not currently a Chinese Version.

Thus, the current study is aimed to investigate whether OBQ-44 is reliable and valid in Chinese population. We translated it into Chinese, and then we validate its psychometric properties in non-clinical and clinical samples. The availability of Chinese OBQ-44 would provide Chinese researchers with a valid measurement to evaluate the severity of dysfunctional beliefs in Chinese context, and to facilitate cross-cultural comparison in the future.

## Methods

### Participants

The non-clinical sample was made up of five hundred and sixty-nine undergraduate volunteers recruited from Sun Yat-Sen University in Guangzhou, China (female: 521, male: 48). Theirs ages ranged from 17 to 23, and the average age was 19.79 (SD = 1.68). Sixty-six OCD patients were the out-patients of Guangzhou Psychiatry Hospital. Nineteen (28.8 %) of participants were female, and forty-seven (71.2 %) were male. Theirs ages ranged from 12 to 44, and the average age was 25.15 (SD = 7.98). The average years of their education was 12.56 (SD = 3.29). OCD patients were diagnosed with DSM-IV criteria for OCD by Structured Clinical Interview (SCID) [12]. A subgroup of 371 non-clinical participants and 23 OCD patients were invited to complete a test-retest session four weeks later. An experienced psychiatrist administered all clinical ratings.

Potential non-clinical and clinical participants were excluded if they: (1) were younger than 18 or older than 50; (2) had a history of head injury, central nervous system diseases, or mental illness (except OCD patients); (3) had a history of substance abuse. These exclusion criteria ensured that all the participants could understand the procedures in the study.

### Procedure

The present study was approved by the ethics committee of Guangzhou Psychiatry Hospital. Written consent was obtained from each study participant before the survey, and the confidentiality of the data use was ensured. Then the participants were instructed to complete the questionnaires. The non-clinical samples were undergraduate volunteers. They completed the questionnaires in the classroom at one time. The clinical samples were OCD patients and they completed the questionnaires in the hospital one by one. All research assistants were very well trained in administering the instruments before the survey.

### Measures

#### The Chinese version of OBQ-44

OBQ-44 is a self-reporting questionnaire including 44 items to evaluate the belief domains associated with OCD. The revised version has three subscales: responsibility/threat estimation (RT) with 16 items (1, 5, 6, 8, 15, 16, 17, 19, 22, 23, 29, 33, 34, 36, 39, 41), importance/control of thoughts (ICT) with 16 items (2, 3, 4, 9, 10, 11, 12, 14, 18, 20, 25, 26, 31, 37, 40, 43), and perfectionism/certainty (PC) with 12 items (7, 13, 21, 24, 27, 28, 30, 32, 35, 38, 42, 44). The respondents are requested to score himself or herself what degree the situation described in each particular statement by a seven-point scale (1 = disagree very much; 7 = agree very much). The total score ranges from 44 to 308.

The validation of the Chinese version of OBQ-44 followed the international guidelines suggested by Beaton for cross-cultural validation of self-reported measures: (1) the initial translation of the original scale into the used language; (2) the synthesis of conceptions; (3) the back-translation; (4) the expert committee’s review on the relevance and representation of items used for the final outcome setting, and (5) the piloting of testing and probing to get at understanding of item [13]. After having received authorization from the author of the instrument, two bilingual psychiatrists who had never seen the original scales to ensure their impartiality translated it into Chinese. Then, the two different translations of were compared and merged, and an initial Chinese version of OBQ-44 was born. Later, twenty five OCD in-patients were asked to complete it aimed to verify if patients could understand the various items of the questionnaires. At this time, all suggestions provided by the patients were taken into account and the adjustments were made wherever necessary. Once all items in the Chinese version were considered adequate to use, they were back-translated into English by another bilingual psychiatrist who was not involved in the previous translation process. This second English version OBQ-44 was compared to the original English version in order to identify whether each item is equivalent in meaning to the original.

#### Yale-Brown Obsessive Compulsive Scale (Y-BOCS)

The Chinese version of Y-BOCS was used to assess the severity of OCD symptoms and to provide measurements of concurrent validity of the translated OBQ-44. Y-BOCS includes 10 items, scoring on a 5-point Likert scale ranging from 0 (disagree very much) to 4 (agree very much). The first five items are summed to provide an obsession severity score (OS) and the last five items shows the compulsions severity score (CS). The Chinese version of Y-BOCS has excellent convergent and divergent validity; the retest reliability and internal consistency are satisfactory [14]. Cronbach’s alpha for Y-BOCS is 0.90 in non-clinical sample and 0.80 in clinical sample, respectively, in this study.

#### Beck Depression Inventory (BDI)

The 21-item BDI was used in present study to assess the severity of depression symptoms over the past two weeks and to provide measurements for the concurrent validity of the translated OBQ-44. Responses were provided on 4-point Likert scale ranging from 0 (symptoms not present) to 3 (symptom strongly present). The Chinese version of BDI has excellent split reliability and the internal consistency is satisfactory [15]. Cronbach’s alpha for BDI is 0.87 in non-clinical sample and 0.90 in clinical sample, respectively, in this study.

### Data analysis

Data were sorted and analyzed using SPSS 20.0 and AMOS 7.0.We performed a confirmatory factor analysis in the non-clinical sample to test three-factor structure of the Chinese version of OBQ-44 by AMOS 7.0. The goodness-of-fit indexes used in the present study as follow: *χ*^2^/d.f, Comparative Fit Index (CFI), Root Mean Square Error of Approximation (RMSEA), Goodness of Fit Index (GFI) and Normed Fit Index (NFI). We used the criteria recommended by Hu and Bentler [16] for goodness-of-fit indexes in the present study. The criteria are as followed: *χ*^2^/d.f ≤ 3, CFI ≥ 0.95, RMSEA ≤ 0.08, GFI ≥ 0.90 and NFI ≥ 0.90. The Cronbach’s alpha and student’s *t*-test for paired samples were used respectively to assess the internal consistency and the test-retest reliability. Moreover, the convergent validity of the Chinese version was also assessed by Pearson correlations with Y-BOCS and BDI respectively, and the comparison between the score of non-clinical sample and that of clinical sample was also conducted. Two-tailed significance level of 0.05 was considered for all statistical procedures in this study.

## Results

### Confirmatory factor analysis

**Fig. 1:**
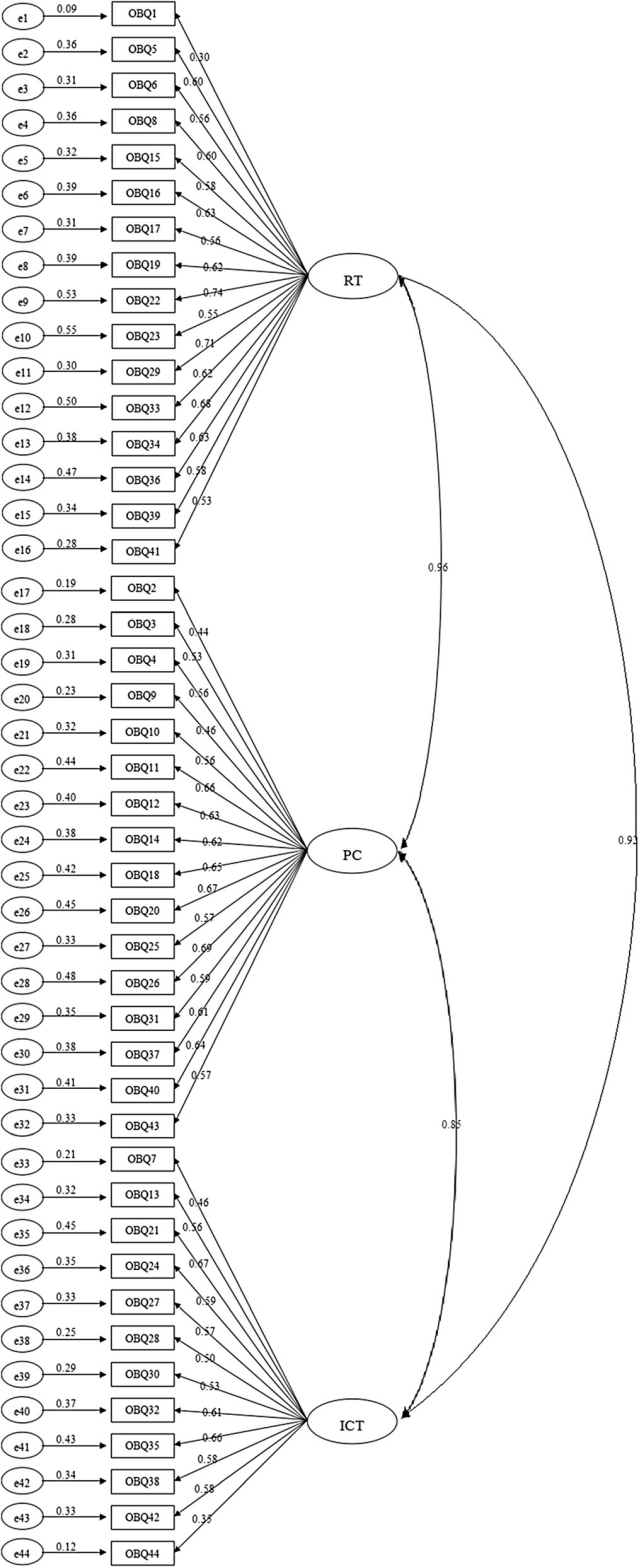
Completely standardized factor loading from the confirmatory factor analysis

We performed a CFA to test the applicability of OBQ-44 to Chinese culture. In our study, maximum likelihood estimation method on the covariance matrix was used to estimate the model fit. The results from CFA suggested an acceptable fit for the model: *χ*^2^/d.f = 2.96, GFI = 0.83, NFI = 0.82, CFI = 0.88, RMSEA = 0.06 and SRMR = 0.06. Figure 1 pretended the path diagram and standardized factor loading for each items.

#### Reliability statistics

The internal consistency and test-retest reliability of the instrument were calculated respectively for the non-clinical group and clinical group (see Table 1).

**Table 1:** Internal consistency of OBQ-44 (total and subscales scores)

	Non-clinical sample (*n* = 569)	Clinical sample (*n* = 66)
OBQ-44	0.954	0.962
RT	0.902	0.930
PC	0.899	0.917
ICT	0.840	0.869

*OBQ*-*44* Obsessive Beliefs Questionnaire 44-item version, *RT* Responsibility/Threat estimation, *PC* Perfectionism/Certainty, *ICT* Importance/ Control of Thoughts

In non-clinical group (*n* = 569), Cronbach’s alpha for overall scale and three subscales are listed as follows: overall OBQ-44, α = 0.954; RT, α = 0.902; PC, α = 0.889; ICT, α = 0.840. For the clinical group (*n* = 66), Cronbach’s alpha for overall scale and three subscales are listed as follows: overall OBQ-44, α = 0.962; RT, α = 0.930; PC, α = 0.917; ICT, α = 0.869. All revealed a very good consistency.

For the non-clinical sample (*n* = 371), the coefficients of test-retest correlation are 0.709 for RT, 0.729 for PC, 0.635 for ICT, and 0.786 for the whole scale. Moreover, for the clinical sample (*n* = 23), the coefficients of test-retest correlation are 0.752 for RT, 0.745 for PC, 0.653 for ICT, and 0.723 for the scale. All test-retest correlations were significant, which indicates OBQ-44 has adequate levels of stability for a measurement.

#### Validity statistics

The convergent validity of OBQ-44 was assessed by calculating the correlations between OBQ-44 scores (total and subscales) and the scores of Y-BOCS and BDI in both non-clinical sample and clinical sample (Table 2). If the correlation between OBQ-44 scores and Y-BOCS scores (indicating the severity of OCD symptoms) was significant, and was stronger than the correlation between the OBQ-44 scores and the BDI scores (indicating the severity of depression), OBQ-44 would show a good convergent validity. According to Table 2, the correlations between the OBQ-44 scores and the other measurements (Y-BOCS and BDI) were all significant in two samples. The convergent validity of OBQ-44 was good with the OBQ-44 scores correlated stronger with Y-BOCS than BDI in non-clinical sample. However, the correlations between the OBQ-44 scores (total and subscale scores) and the Y-BOCS scores were significant and showed a tendency to be lower than the correlation between the OBQ-44 scores (total and subscale scores) and the BDI scores in clinical sample. Moreover, the correlations between the OBQ-44 scores and the Y-BOCS total scores partialling out BDI scores was 0.358 (*P* < 0.01) in non-clinical sample, and 0.312 (*P* = 0.012 < 0.05) in clinical sample. These results showed the OBQ-44 had an acceptable convergent validity. Table 3 shows that OCD patients demonstrated significantly higher OBQ-44 total scores and subscale scores than non-clinical samples.

**Table 2:** Correlations between OBQ-44 (total and subscales scores) and Y-BOCS, and BDI

	OBQ-44	RT	PC	ICT
Non-clinical sample (*n* = 569)				
Y-BOCS	0.431^**^	0.422^**^	0.375^**^	0.403^**^
OS	0.405^**^	0.404^**^	0.349^**^	0.376^**^
CS	0.401^**^	0.384^**^	0.353^**^	0.374^**^
BDI	0.335^**^	0.349^**^	0.292^**^	0.290^**^
Clinical sample (*n* = 66)				
Y-BOCS	0.419^**^	0.402^**^	0.412^**^	0.312^*^
OS	0.265^*^	0.230^*^	0.280^*^	0.206^*^
CS	0.334^**^	0.344^**^	0.311^*^	0.240^*^
BDI	0.515^**^	0.537^**^	0.432^**^	0.437^**^

*P < 0.05, **P < 0.01

*OBQ-44* Obsessive Beliefs Questionnaire 44-item version; *RT* Responsibility/Threat estimation; *PC* Perfectionism/Certainty; *ICT* Importance/Control of Thoughts; *YdBOCS* the Yale-Brown Obsessive-Compulsive Scale; *OS* Obsessions severity; *CS* compulsions severity; *BDI* the Beck Depression Inventory

**Table 3:** Means and Standard Deviations for subscales and total scores

	Clinical sample (*n* = 66)	Non-clinical sample (*n* = 569)	*t*
RT	65.89 (22.97)	39.27 (14.14)	9.23^**^
PC	65.50 (22.01)	42.24 (14.72)	9.81^**^
ICT	42.85 (15.87)	25.65 (9.60)	8.62^**^
Total score	178.23 (55.51)	107.19 (35.80)	10.15^**^

^*^ *P* < 0.05, ^**^ *P* < 0.001

## Discussion

The purpose of this study was to examine the psychometric properties of Chinese version of OBQ-44 in both clinical and non-clinical samples. In our non-clinical sample, a confirmatory factor analysis confirmed the three-factor structure of the OBQ-44, namely responsibility/threat estimation (OBQ-RT), perfectionism/certainty (OBQ-PC), and importance/control of thoughts (OBQ-ICT). These results indicated that Chinese version of OBQ-44 is consistent to the model of the original version [5], Brazilian [10], German [8], and Arabic versions [9], and inconsistent to the model of Iranian version [11]. In Shams’ study, the exploratory factor analysis was carried out, and the results indicated Persian language version of the OBQ-44 had five factors. The differences in the number of factors may be explained by the difference between the confirmatory factor analysis and exploratory factor analysis, because items are assigned to a single factor in a confirmatory factor analysis, but they tend to load on more than one of factor in exploratory factor analysis [17]. Moreover, all items in the measurement had loading greater than (or equal to) 0.30 in our study. Based on these results, the Chinese version of OBQ-44 is suggested to have good construct validity.

An analysis of the whole scale and the three subscales for non-clinical samples demonstrated the Chinese version of OBQ-44 have high internal consistencies as indicated by the Cronbach’s alpha coefficients, which is consistent with the results reported by Julien [7], and slightly higher than those results reported by Rahat [9]. Test-retest reliability with 4-week interval (0.709 for RT, 0.729 for PC, 0.635 for ICT, and 0.786 for the whole scale) were assessed and considered acceptability, slightly lower than those results reported by Rahat [9].

For clinical samples, the Cronbach’s alpha coefficients of 44 items and three subscales showed a very good internal consistency for the Chinese version of OBQ-44, which is consistent with those reported by Bortoncello [10]. Moreover, clinical samples had higher Cronbach’s alpha coefficients than the non-clinical sample in current study. This might be due to the different numbers between non-clinical (*n* = 569) and clinical samples (*n* = 66). Although the Chinese version shows less test-retest reliabilities compared to that of the French version [7], the test-retest reliabilities range from moderate to acceptable values. Test-retest of the Chinese version in our study suggests that it shows an acceptable stability. The inconsistent might be due to the longer interval time between test and retest in our study (interval time: 4 weeks) than that in Julien’s study (interval time: 3 weeks) [7].

As expected, zero-order correlations between the OBQ-44 scores (total and subscale scores) and the other clinical measures i.e. Y-BOCS, and BDI were significant. There were partial support for the correlations between the OBQ-44 scores (total and subscale scores) and the Y-BOCS were significant and the correlations showed a tendency to be stronger than the correlation between the OBQ-44 scores (total and subscale scores) and BDI in non-clinical sample. However, in the clinical sample the correlations between the OBQ-44 scores (total and subscale scores) and the Y-BOCS showing a tendency to be lower than the correlation between the OBQ-44 scores (total and subscale scores) and BDI. Julien *et al*. [7] also reported the low correlations between the OBQ-44 scores (total and subscale scores) and the Y-BOCS in clinical sample. Thus, the convergent validity of the Chinese version of OBQ-44 needs further research.

A major strength of the present study is that non-clinical and clinical samples were used to the validation of the Chinese version of OBQ-44. However, there are several limitations to be considered. First, the clinical sample is relatively small, and all the participants in the non-clinical sample were undergraduates, and female participants were over represented in the non-clinical sample, which may limit the generalizability of our finding. Psychometric analyses should be carried out in a more large and representative sample. Moreover, because the divergent validity of the Chinese version was only determined by the comparison of the OBQ-44 score of the OCD patients and of the undergraduates, and did not assess the anxiety level to show divergent validity. This is another limitation of this study. Finally, more information about the Chinese version’s the correlations with multiple scales of OCD symptoms should be used to examine its convergent validity.

## Conclusion

The results of the current study suggest that the Chinese version of OBQ-44 is a reliable and valid instrument to assess dysfunction beliefs in Chinese population. It is also useful for researchers to evaluate interventions aiming to reduce dysfunction beliefs. Furthermore, its validity is contributed to the cross-cultural comparison in the future.

## Abbreviations

BDI: Beck Depression Inventory
CFI: Comparative fit index
DSM- IV: Diagnostic and Statistical Manual of Mental Disorders Criteria, fourth edition
GFI: Goodness of Fit Index
ICT: Importance/Control of Thoughts
NFI: Normed Fit Index
OBQ-44: Obsessive Beliefs Questionnaire-44
OCCWG: Obsessive Compulsive Cognitions Working Group
OCD: Obsessive-Compulsive disorder
PC: Perfectionism/Certainty
RMSEA: Root Mean Square Error Approximate
RT: Responsibility/Threat Estimation
SD: Standard Deviation
Y-BOCS: Yale-Brown Obsessive-Compulsive Scale
